# Supplementary material for: Early life-stage thermal resilience is determined by climate-linked regulatory variation
Source: Proc Natl Acad Sci U S A. 2026 Jan 8;123(2):e2518358123. doi: 10.1073/pnas.2518358123 (PMC12799179; doi:10.1073/pnas.2518358123)
Supplement: Supplementary file 1 — Appendix 01 (PDF) [file pnas.2518358123.sapp.pdf]

PNAS

Supporting Information for
Early life-stage thermal resilience is determined by climate-linked regulatory variation

Joaquin C. B. Nunez*, Sumaetee Tangwancharoen[†], Kylie M. Finnegan[‡], Eliza M. Bufferd[§], Olin C. King[§], Luke A. Proud, and Brent L. Lockwood*

Dept. of Biology, University of Vermont; Burlington, Vermont, USA
*Corresponding authors: Brent.Lockwood@uvm.edu, Joaquin.Nunez@uvm.edu

† Current address: Chulalongkorn University; Bangkok, Thailand
‡ Current address: Dept. of Organismic and Evolutionary Biology, Harvard University; Cambridge, MA, USA
§ Equal contribution

The PDF file includes:

Figure S1: Embryonic heat tolerance (LT_{80}) of F16 samples.
Figure S2: Coverage and nucleotide diversity for pooled samples.
Figure S3: Genomic similarity between the F16 samples and the VT and SK parents.
Figure S4: Sliding window analysis for F_{ST} outliers in the introgression test.
Figure S5: Patterns of allele frequency from Pool-Seq and individual sequencing data.
Figure S6: The top hits in the X chromosome and embryonic phenotypes.
Figure S7: Patterns of allele frequency introgression in the region in the X chromosome.
Text S1: Details on the GO enrichment analysis.
Text S2: The evolutionary context of our top SNPs in 2R and X.

Supplementary Datasets:

Dataset S1: Sample metadata.
Dataset S2: Top SNPs in Fisher's exact test.
Dataset S3: Gene ontology (GO) analyses in 2R.
Dataset S4: Gene ontology (GO) analyses in X.
Dataset S5: Gene ontology (GO) analyses genome-wide.
Dataset S6: Embryonic heat shock survival data in the DGRP
Dataset S7: Developmental gene expression data for genes related to the top SNP in 2R.
Dataset S8: Regulatory SNP annotations associated with *SP70* and *sog*.
Dataset S9: Allele age estimates (https://doi.org/10.5281/zenodo.17059130).

Figure S1: Embryonic heat tolerance (LT_{80}) of F16 samples.

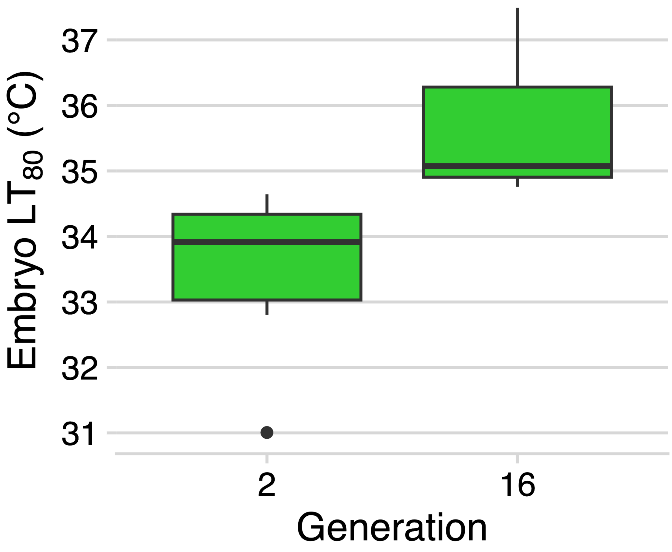

Embryonic heat tolerance (LT_{80}) estimated from the exposure (1 hour at the indicated temperature) that induced 80% mortality among six replicate F2 and F16 introgression lines.

Figure S2: Coverage and nucleotide diversity for pooled samples.

A

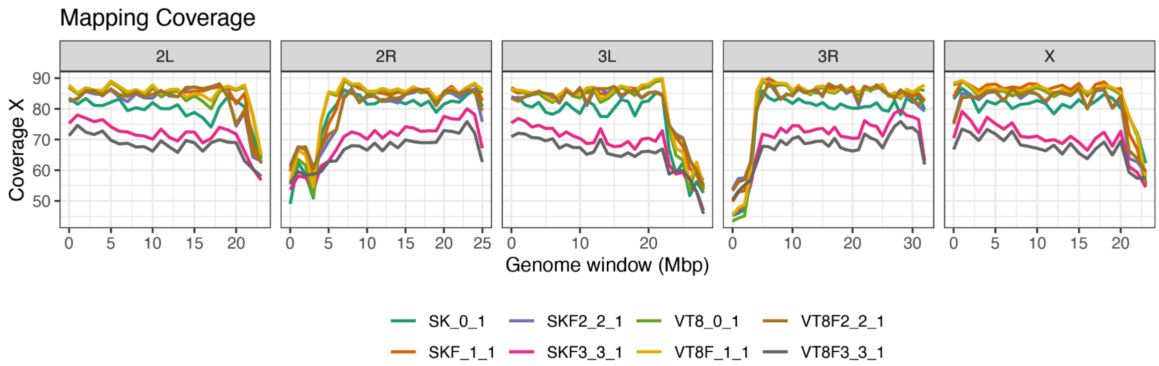

B

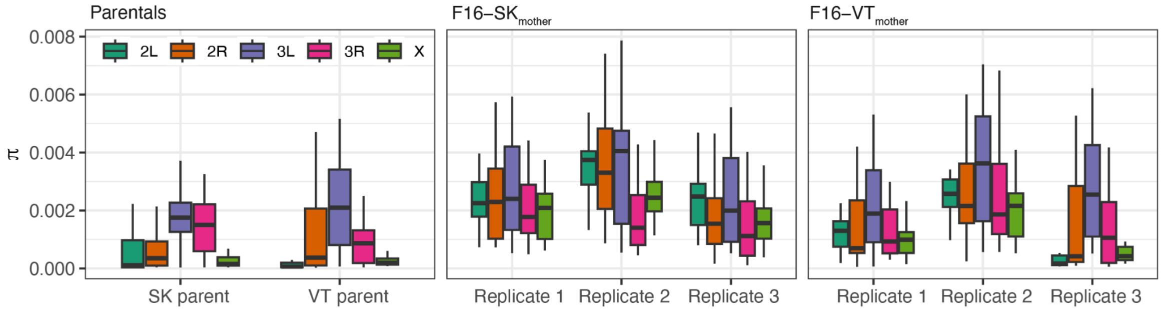

(A) Coverage across chromosomes of *D. melanogaster* for all pools. To investigate the genomic basis of this response, we performed pooled whole-genome sequencing on six replicate F16 introgressed populations: in three, VT was the maternal parent, and in the other three, SK was the maternal parent. Sequencing was also performed on the original VT and SK parental strains. Sequencing results of both the parental pools and the six F16 introgression pools have deep levels of coverage (mean = 87.6X, sd = 14.1X) and high mapping quality scores (mean = 56.6, sd = 0.249). Considered individually, the parental lines and the replicate pools 1 and 2 for F16 crosses have similar coverages (78X-82X). Pools from the third F16 replicate have slightly lower, yet deep, levels of coverage (66X-69X). Given pools were sequenced on individual flow cells, these coverage differences suggest a mild technical effect for the third replicate of our crosses. Yet, this is not a concern for downstream analyses since our analytical tools account for coverage differences by design (see materials and methods). The estimated levels of PCR and optical duplicates filtered during mapping was ~0.25% (sd = 0.03%). Combined, these results indicate that our sequencing efforts yielded high-quality data. The panels show the mean coverage across each chromosome. The colors, indicated in the legend, are each replicate pool. Parental lines are labeled as “0_1” (e.g., SK_0_1 and VT8_0_1), and replicates of the F16s are labeled as “n_1” where n is the replicates (e.g. , 1, 2, 3). **(B)** Levels of nucleotide diversity (**π**) across chromosomes for parental lines and all F16 pools, contingent on whether SK or VT was used as the mother in the cross. After applying a 5% minimum threshold for missing data, we discovered 865,020 SNPs segregating among all pools. Of these, 159,641 are found in 2L, 205,515 in 2R, 187,745 in 3L, 203,632 in 3R, and 108,859 in X. Analyses of nucleotide diversity (π) showed lower π in our parental lines (ANOVA; $F_{2,1085}$ = 63.12, P = 2.0x10^{-16}) relative to our introgressed lines. For example, parental inbred lines in VT and SK had **π** levels ranging from 0.10-0.11. In contrast, F16 lines had doubled the level of π (i.e., 0.239 and 0.186; for the F16 initiated with SK-mothers and VT-mothers, respectively). These data revealed two salient results: first, the SK and VT parental lines harbor residual heterozygosity. Second, 7 generations of introgression did not return π to the original levels of the parental VT lines, a likely byproduct of the segregating residual heterozygosity from the initial SK x VT cross.

Figure S3: Genomic similarity between the F16 samples and the VT and SK parents.

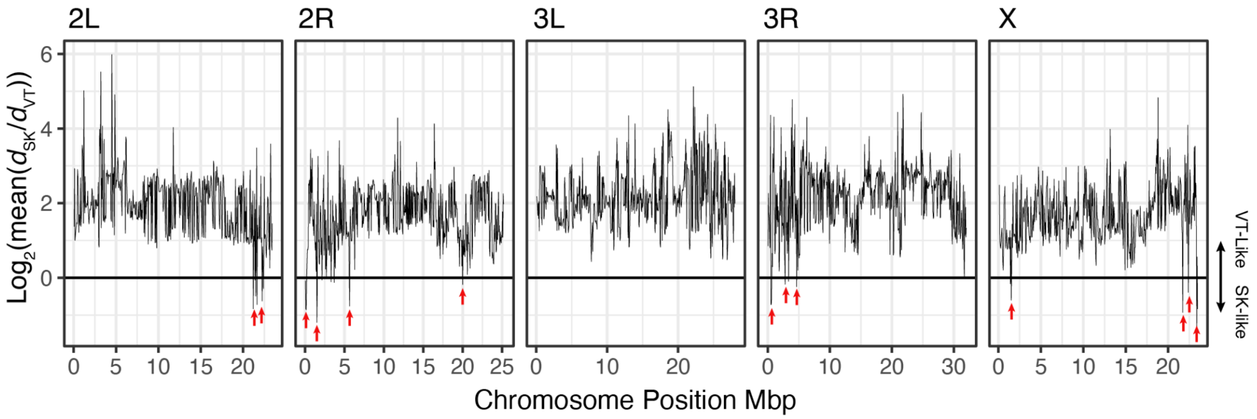

To investigate whether particular regions of the F16 genomes show differential patterns of introgression, relative to the parental lines, we estimated local PCAs using a sliding window approach (window size = 0.1 Mb; step = 50 Kb). We then estimated the mean euclidean distance (i.e., *d*), using PCs 1 and 2, across F16 pools relative to each parent (i.e., d_{SK} and d_{VT}). Accordingly, the ratio of d_{SK} and d_{VT} is a proxy for genetic distance, whereby $\mathrm{Log}_2(d_{SK}/d_{VT}) > 0$ indicates higher similarity to the VT parent and $\mathrm{Log}_2(d_{SK}/d_{VT}) < 0$ indicates higher similarity to the SK parent.

Figure S4: Sliding window analysis for F_{ST} outliers in the introgression test.

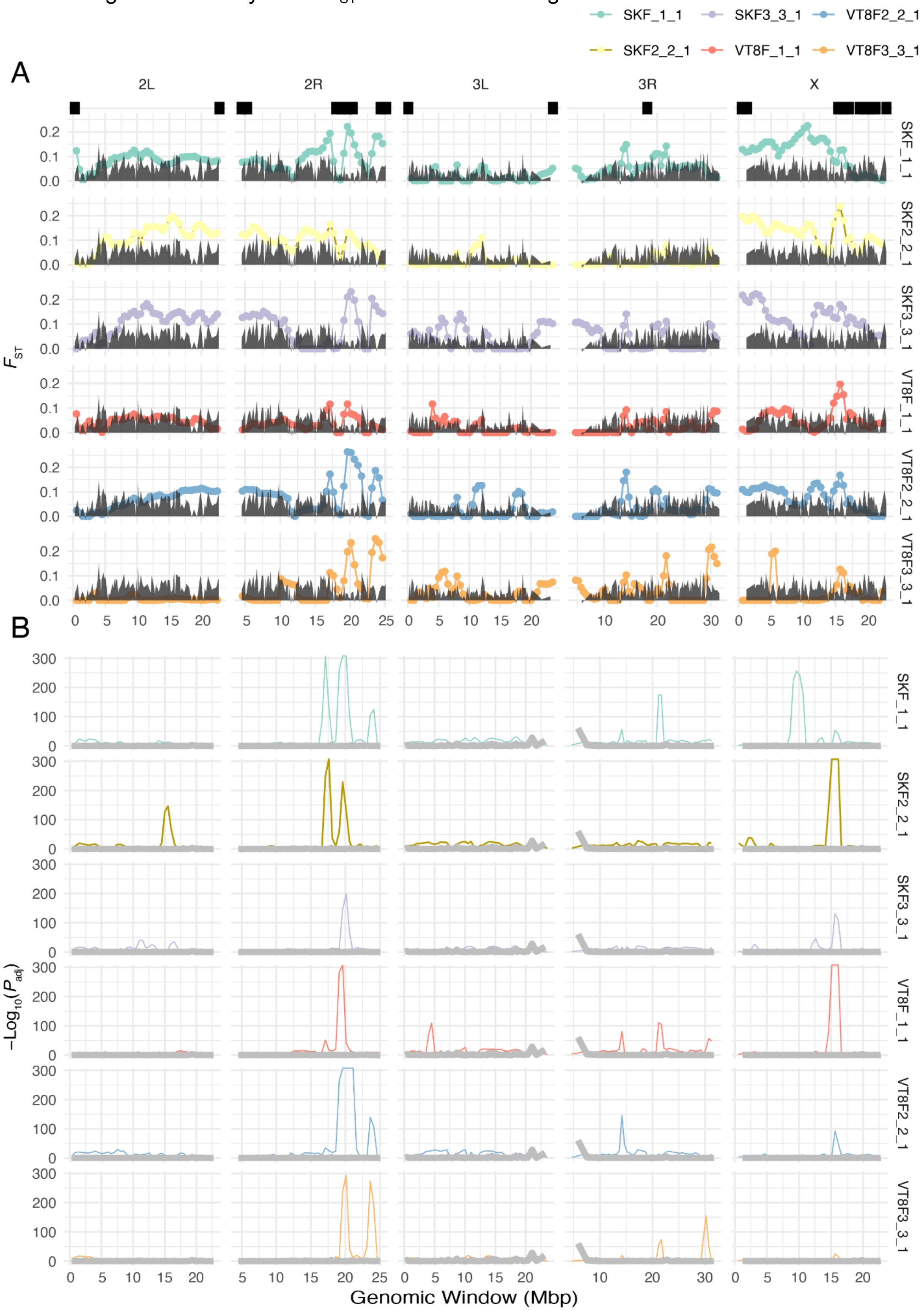

(A) Sliding window analysis for F_{ST} outliers across our introgression crosses relative to neutral introgression simulations across all chromosomes. **(B)** Sliding window analysis for the enrichment of F_{ST} in the top 1% of the distribution across the genome.

Figure S5: Patterns of allele frequency from Pool-Seq and individual sequencing data.

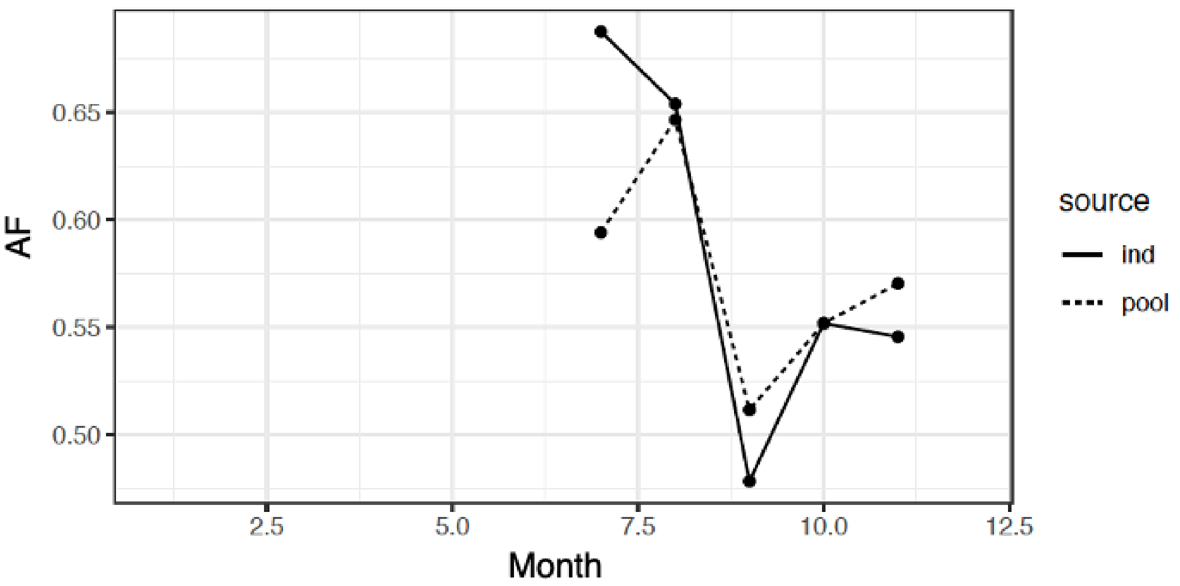

Patterns of allele frequency for 2R:20,551,633 from Pool-Seq (dashed lines) and individual (solid lines) sequencing data. Allele frequency estimates from pooled sequencing (DEST Virginia samples) are shown alongside individually sequenced flies from Nunez et al. (2024), collected at the same site in 2016. Patterns of allele frequency change are concordant between datasets (Granger Causality Test, P = 0.032).

Figure S6: The top hits in the X chromosome and embryonic phenotypes.

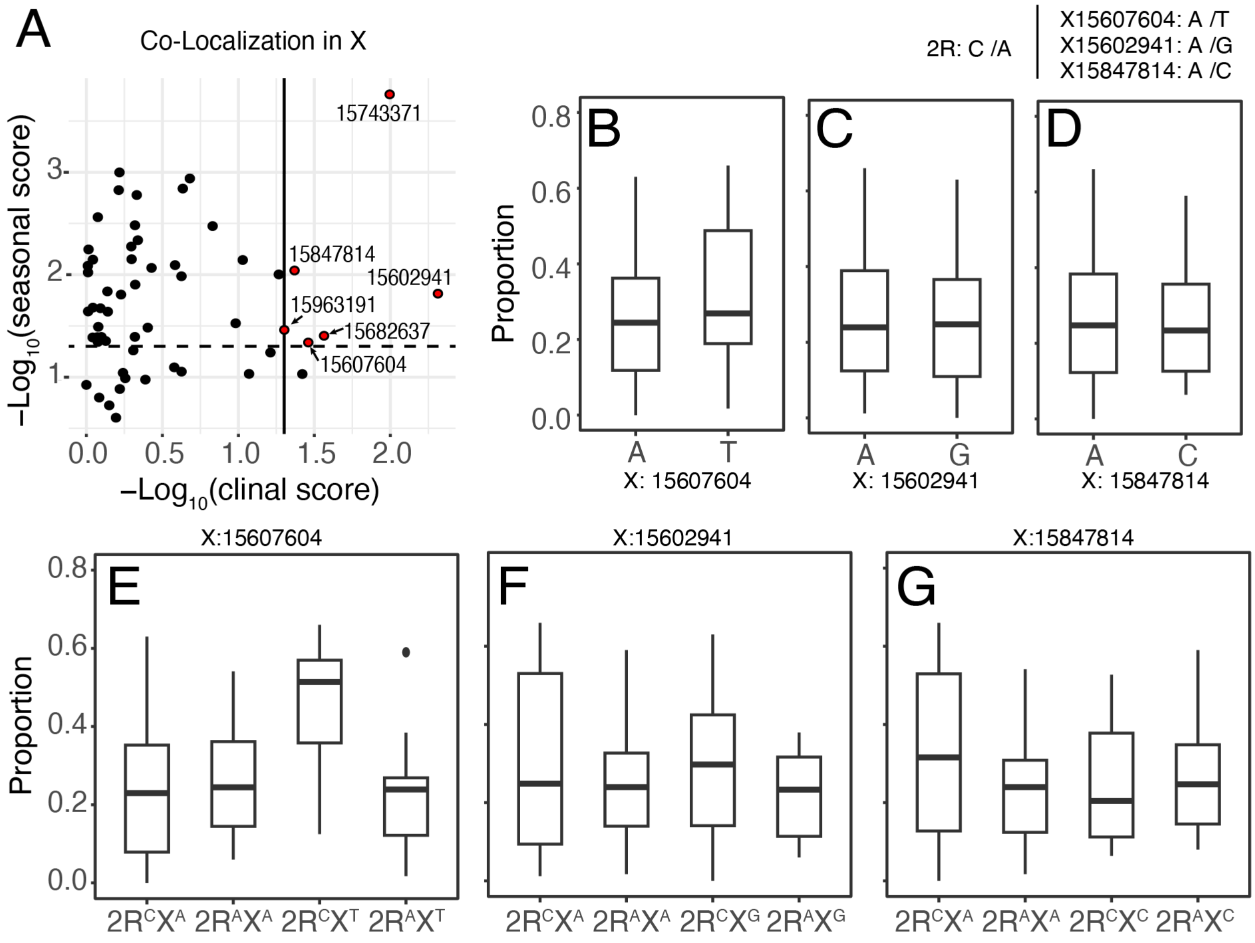

(A) SNPs that are jointly FET outliers, seasonal outliers, and clinal outliers in the window of interest of the X chromosome. **(B-D)** Embryonic survival for genotypes across the top three SNPs in the X chromosomes. **(E-G)** Embryonic survival for genotype combination across the top hit in 2R and the three top SNPs in the X chromosomes. The only combination with a significant effect, $2R^CX^T$, shown in panel E, is the same combination highlighted in the main text as the "tropical genotype."

Figure S7: Patterns of allele frequency introgression in the region in the X chromosome.

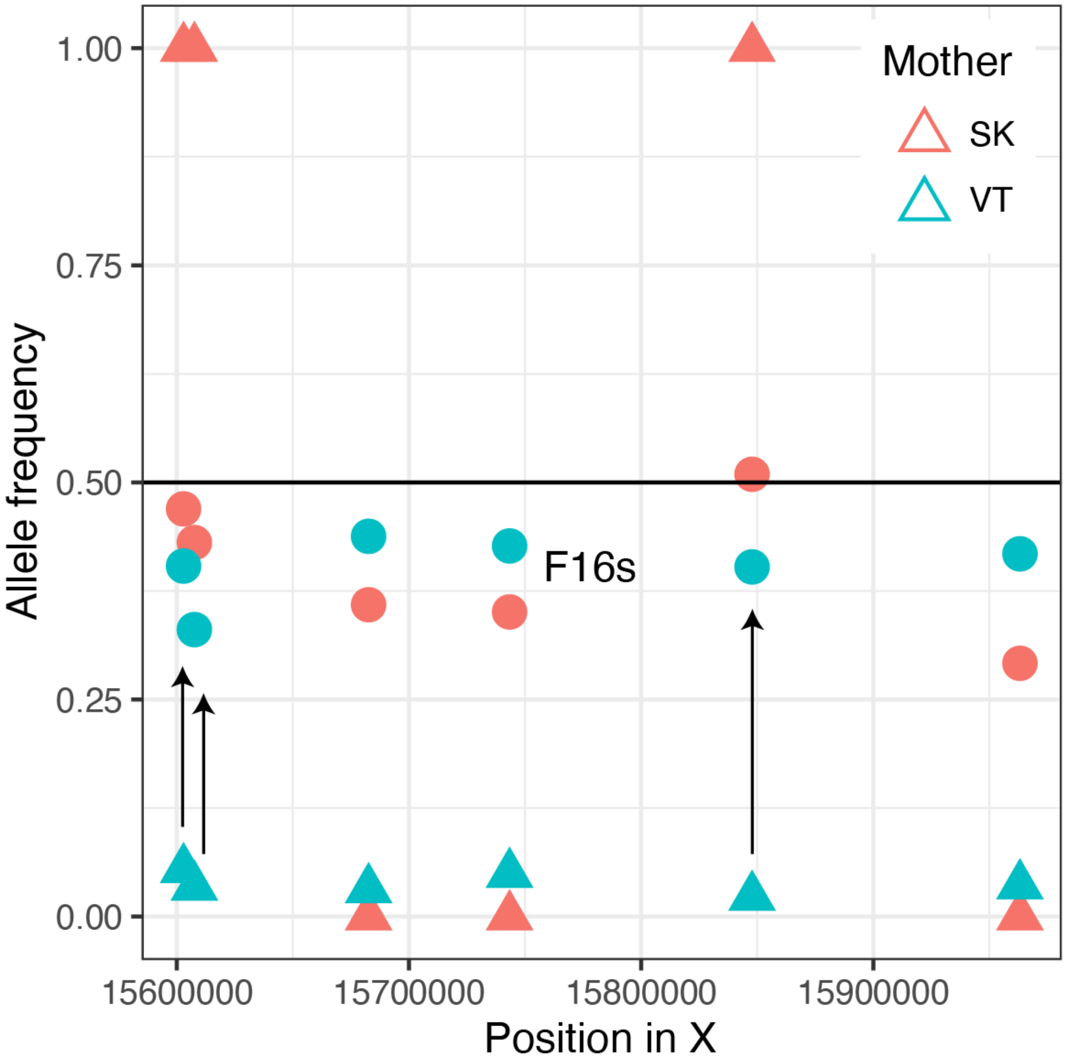

Patterns of allele frequency introgression in the region of interest of the X-chromosome. The red triangles indicate the allele frequency in the SK background. The blue triangles indicate the allele frequency in the VT background. The circles indicate the allele frequency of the F16 offspring, the color of the circle indicates the background of the mother. SNPs showing patterns consistent with adaptive introgression (i.e., towards the SK background) are indicated with arrows.

Text S1: Gene Ontology Enrichment analyses

We calculated GO enrichment scores using the outlier SNPs, derived from the synthetic pool FET, harbored inside of the windows of interest in 2R and X. For the case of the window in 2R, we observe one GO term with a *Q*-value threshold below 0.1. The term (GO:0005549), is associated with odorant binding functions (*Q* = 0.081) and it is driven by the presence of outlier SNPs at three paralogs of the Odorant-binding proteins 56 and 57 (*Obp56/57*) gene (*Obp56d*, *Obp57g*, *Obp57d*). Two other GO terms in the enrichment are related to heat shock protein (*Hsp70* and *Hsp90*) binding, GO:0030544 and GO:0051879. These terms have low *P*-values (*P*-value = 0.003, *P*-value = 0.005, respectively) but do not pass *Q*-value filtering (*Q*-value > 0.1) in our analysis. Yet, these terms appear in the analysis due to the presence of the *CG15120* gene (FBgn0034454). A gene predicted to play a role in *Hsp70*, *Hsp90*, and ubiquitin protein ligase binding activity.

For the window in the X chromosome, we observed 10 GO terms enriched with a *Q*-value threshold below 0.1. Two of these terms are associated with the regulation of euchromatin (GO:0005719, GO:0000791; *Q*-values of 0.013 and 0.062, respectively) and are driven by two genes: Myb oncogene-like (*Myb*) and DNA oxidative demethylase (*AlkB*). Six are related to t-RNA ligase activity, tRNA aminoacylation, amino acid activation, as well as other functions related to carbon-oxygen bonds (GO:0016876, GO:0016875, GO:0006418, GO:0004812, GO:0043039, GO:0043038; *Q*-values = 0.062 throughout). Two genes primarily drive the enrichment of these terms, *CG8097*, which encodes the arginine--tRNA ligase enzyme, and the Arginyl-tRNA synthetase (*ArgRS*) gene. The last two terms are related to centriole assembly and replication (GO:0007099, GO:0098534; *Q*-values = 0.068 for both). These terms are driven by SNPs found in the aforementioned *Myb*, as well as of the Chaperonin containing TCP1 subunit 6 (*CCT6*) gene.

Text S2: The evolutionary context of our top SNPs in 2R and X

To better understand the findings from our adaptive introgression experiment, we integrated findings from ecological genomics analyses using the DEST dataset. Through this approach, we identified two high-confidence SNPs within our focal regions (2R:20,551,633 and X:15,607,604). In the case of 2R, the tropical allele ("C") is the ancestral allele relative to four outgroup species (*D. simulans, D. yakuba, D. sechellia, and D. erecta*) and it is the major allele in Africa. For the case of X, the tropical ("T") is the ancestral allele relative to *D. simulans,* and *D. sechellia*; notably, *D. yakuba* shows a different allele altogether ("G").

While both mutations showed a response to selection in our experiment, we did not detect any meaningful linkage disequilibrium (LD) between them in VA ($r^2_{X\text{-}2R\ SNPs}$ = 0.0008) or in the DGRP panel ($r^2_{X\text{-}2R\ SNPs}$ = 1.232×10^{-5}). For the particular case of our 2R candidate, given the close physical proximity of our top SNP to the cosmopolitan inversion *In(2R)NS*, we investigated the extent of potential linkage between these loci. To this end, we calculated LD within a genomic window of approximately 10^7 bp in the DGRP panel, including a set of established inversion markers for *In(2R)NS* (see materials and methods). The results showed that, despite its physical proximity to the inversion, the top SNP in 2R segregates independently of *In(2R)NS* (**Fig. TS2-1**).

We also inferred the relative ages of the mutation and the inversion using a phased dataset from wild Virginia populations (**Dataset S9**). Our results suggest that both the 2R and X mutations are old ($TMRCA_{2R\ SNP}$= 214,384.8 ya; $TMRCA_{X\ SNP}$= 151,074.8 ya), predating the most recent out-of-Africa migration by at least 140,000-200,000 years. In the particular case of 2R, the top SNP emerged roughly three times earlier than the *In(2R)NS* inversion (Mean $TMRCA_{inv.\ markers}$ = 62,303.9 ya).

Figure TS2-1:

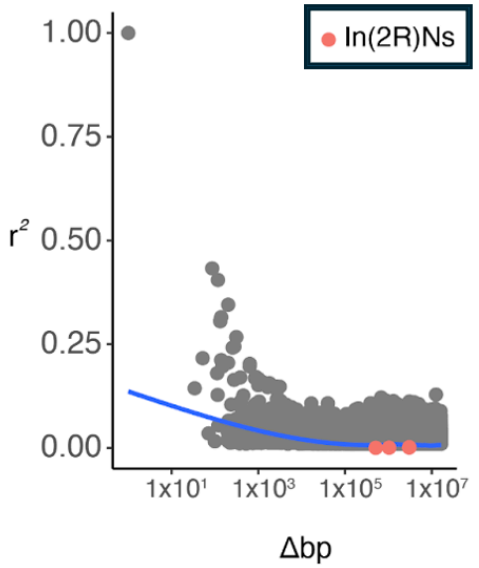

Patterns of linkage disequilibrium (r^2) in the window of interest in chromosome 2R, in relationship to the top SNP in 2R. The in(2R)NS markers are shown in red.
